# Defining Key Features of Complex Coronary Lesions: An Evidence Based Review of Clinical Practice. Part II: Chronic Total Occlusions, Graft Interventions, In-Stent Restenosis, and Antithrombotic Strategies

**DOI:** 10.31083/j.rcm2306209

**Published:** 2022-06-08

**Authors:** Daniel Feldman, Frans Beerkens, Johny Nicolas, Mohan Satish, Davis Jones, Mehmet Demirhan, George Dangas

**Affiliations:** ^1^The Zena and Michael A. Wiener Cardiovascular Institute, Icahn School of Medicine at Mount Sinai, New York, NY 10029-6574, USA; ^2^Department of Medicine, Icahn School of Medicine at Mount Sinai, New York, NY 10029, USA; ^3^Department of Medicine, Elmhurst Hospital Center, Queens, NY 11373, USA

**Keywords:** complex percutaneous intervention, chronic total occlusion, in-stent restenosis, saphenous vein graft, dual antiplatelet

## Abstract

Clinicians have long recognized that certain features of coronary artery lesions increase the complexity of intervention. Complex lesions are associated with worse cardiovascular outcomes and a higher risk of subsequent ischemic events. These lesions are categorized by their angiographic features. These features include bifurcation lesions, left main coronary artery disease, calcified lesions, in-stent restenosis, chronic total occlusions and graft interventions. This two-part review aims to highlight the current evidence in the percutaneous management of these lesions. Part two of this review focuses on the indications to treat chronic total occlusions, interventions of failed grafts, tools used to treat in-stent restenosis, as well as antithrombotic strategies.

## 1. Chronic Total Occlusion

A chronic total occlusion (CTO) is an occlusion with TIMI (Thrombolysis in Myocardial Infarction) grade 0 flow for greater than 3 months. They can also be classified by the type of collateralization as defined by the Rentop Grading system. In this system Grade 0 has no collaterals, Grade 1 has filling of side branches without visualization of the epicardial segments, Grade 2 has partial filling of the epicardial segment via collateral channels and Grade 3 has complete filling of the epicardial segment via collateral channels [1]. These type of lesions are present in roughly 20% of patients with chronic ischemic heart disease and are frequently well collateralized [2, 3].

Independently, CTOs are associated with a poor prognosis, high mortality rate, high likelihood of ventricular arrhythmias and persistent anginal symptoms [4, 5, 6, 7]. Successful revascularizing of these territories may be associated with improved quality of life (QoL), fewer anginal symptoms or improved left ventricular ejection fraction (LVEF) compared to unsuccessful CTO-PCI (percutaneous intervention), but has not been well-studied versus medical therapy alone without attempting the CTO-PCI [3, 8, 9]. CTO interventions, however, have a rather high rate of complications and relatively low procedural success ranging from 60–90% overtime [2, 10]. Hence, there is broad debate on appropriate indications and management of CTO lesions [11].

### 1.1 Indications for CTO revascularization

Current guidelines for revascularization of CTO lesions are limited. The 2021 ACC/AHA/SCAI guidelines only indicate that it is unknown whether CTO treatment improves anginal symptoms [12]. The 2018 ESC/EACTS guidelines recommend PCI to CTO in patients with regional wall motion abnormalities in areas supplied by CTO if there is evidence of viability and for persisting symptoms [13].

More recently there have been four randomized controlled trials (RCTs) that have evaluated benefits of CTO intervention. The EXPLORE (Evaluating Xience and Left Ventricular Function in Percutaneous Coronary Intervention on Occlusions After ST-Elevation Myocardial Infarction) trial randomized patients with STEMI (ST segment elevation myocardial infarction) who additionally had a CTO to treatment with PCI or medical therapy. This trial found no benefit in LVEF four months after PCI compared to medical therapy, however there was a benefit in the subgroup who received PCI to the CTO of the LAD (left anterior descending artery) [14]. The EURO-CTO (Evaluate the Utilization of Revascularization or Optimal Medical Therapy for the Treatment of Chronic Total Coronary Occlusions) trial thereafter compared CTO-PCI to medical therapy and showed improvement in anginal symptoms and QoL [15]. A secondary analysis showed lower rates of ischemia driven revascularization in the PCI group [15]. The DECISION-CTO (Drug-Eluting Stent Implantation Versus Optimal Medical Treatment in Patients With Chronic Total Occlusion) trial compared CTO-PCI to medical therapy in those with stable angina, acute coronary syndrome (ACS) and silent ischemia. This trial showed no benefit in clinical outcomes associated with CTO-PCI. Notably, however, there was a considerable amount of people in the medical therapy arm that crossed over to the PCI arm. When analysis was performed per protocol the PCI arm out-performed medical therapy alone [16]. Lastly, the REVASC (Recovery of Left Ventricular Function After Stent Implantation in Chronic Total Occlusion of Coronary Arteries) trial compared CTO-PCI to medical therapy and did not find any difference in its primary outcome of changes in regional wall motions or left ventricular function; however secondary outcomes of MACE (major adverse cardiovascular events) at 12 months were lower in the CTO-PCI arm [17].

This literature raises some important questions for management of CTO. The first clinical question is whether revascularization improves clinical outcomes. The EXPLORE trial suggested that it may be beneficial in those with CTO to LAD. The DECISION-CTO and REVASC trials attempted to answer these questions and while neither achieved their primary outcome there is still room for skepticism. The DECISION-CTO trial was underpowered and had a high rate of cross-over. The REVASC trial had limitations because despite its randomization there were differences in the two arms. For instance in the non-CTO revascularization arm there were much higher rates of PCI in coexisting non-CTO segments, particularly in segments that provide collateral blood supply, as study protocol allowed for additional PCI at the time of index diagnostic angiography. Additionally, the study included predominantly healthy ventricles and intervention of CTO segments that do not supply the left ventricle thus any intervention is not expected to have much effect on LVEF.

The second question is whether treating these lesions leads to improved anginal symptoms. It is generally accepted that in the presence of severe symptoms on medical therapy, revascularization is indicated to improve QoL and even subsequent analysis of the ISCHEMIA (International Study of Comparative Health Effectiveness with Medical and Invasive Approaches) trial showed improvement in anginal symptoms and QoL in those who received invasive intervention compared to medical therapy [18]. The EURO-CTO trial provided evidence that treatment with CTO-PCI improves symptom relief. It is worthwhile to consider that this was an open-labeled and unblinded trial and decision to treat a CTO lesion on the basis of symptomatology should also consider coronary anatomy, lesion complexity, viability of distal myocardial beds as well as the risk of complications.

**Fig. 1.**
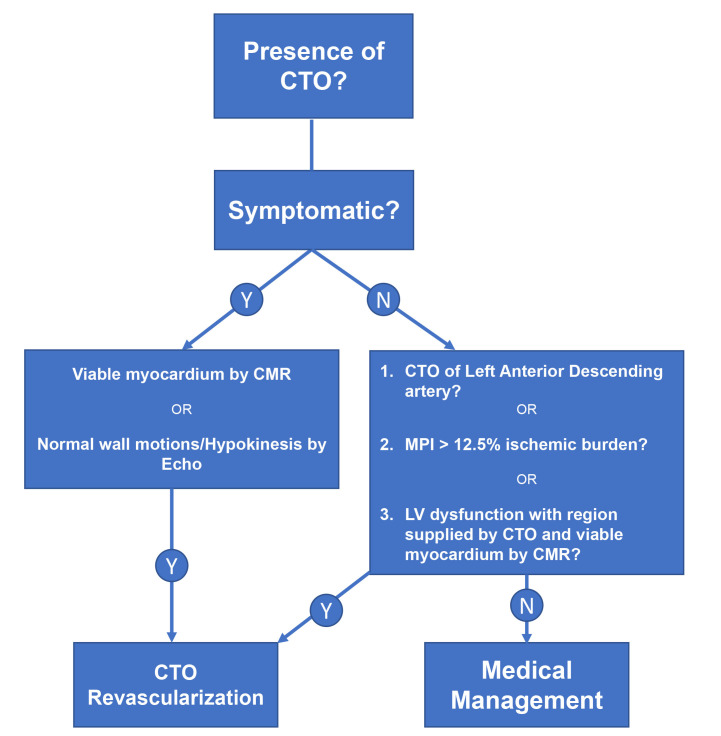
**Algorithm for management of CTO**.

Due to the mixed evidence on CTO interventions algorithms have been developed to guide operators on when to intervene [19, 20, 21]. Generally, revascularization should be considered in the presence of symptoms, if there is expected benefit in LVEF or for ischemic reduction [11]. In the presence of symptoms, either angina or dyspnea, it is important to evaluate for underlying viability. Positive viability testing with cardiac magnetic resonance imaging (CMR) with a threshold of less than 50–75% transmural infarction by late gadolinium enhancement (LGE) has been predictive of functional recovery [22, 23]. In the absence of symptoms, intervention should be attempted if the ischemic burden on MPI (myocardial perfusion imaging) is >12.5% by SPECT/PET/CMR in areas with normal wall motion or hypokinesia by echocardiography [24]. Intervention can also be performed in areas of akinesis or dyskinesis so long as viability is proven even in the absence of symptoms. Additionally, special consideration should be taken for CTO of LAD because of the aforementioned benefit in this group (Fig. 1) [14]. It is also recommended for CTO revascularization to be preferentially performed at high-volume centers [25].

Ultimately, determination of intervention should be patient specific and decided upon by a heart team taking into account anatomy, SYNTAX score, and the patients’ relative comorbidities. Pre-procedural planning is important prior to intervention as certain angiographic and clinical characteristics can help determine if operators should intervene on lesions. Coronary computed tomography angiography may be helpful in determining the CTO length, the presence of calcium and the vessel size [26, 27]. These characteristics have been combined into scoring systems to predict revascularization success. The most widely used of these scoring systems is the J-CTO score where a higher score is predictive of intervention failure [28].

### 1.2 CTO Wiring Techniques

**Fig. 2.**
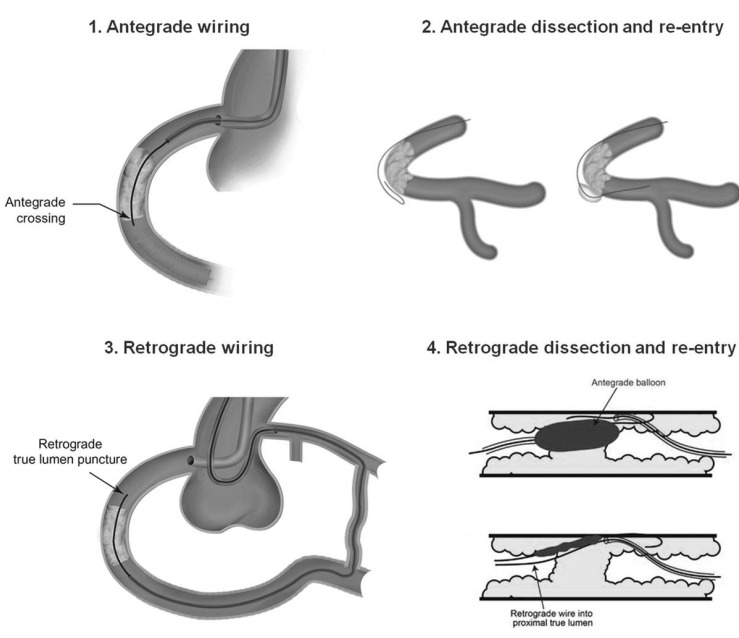
**CTO Wiring Techniques**. Reproduced with permission from Brilakis *et al*. [30]. Copyright © 2019, Wolters Kluwer Health, Inc. **Antegrade**: In the antegrade approach, wires of escalating stiffness are used to cross a CTO. **Retrograde**: In the retrograde approach, the CTO is approached from the distal vessel by advancing a guidewire into the artery distal to the occlusion through a collateral vessel or bypass graft. **Antegrade Dissection & Re-entry**: In the antegrade dissection and re-entry approach, the CTO is approach via the subintimal space followed by re-entry into the true limen distal to the CTO using guidewires or balloon systems.** Reverse Dissection & Re-entry**: In the reverse dissection and re-entry approach the crossing is aided by an angioplasty balloon from the antegrade direction to help make a connection between the two spaces.

Historically the presence of a CTO was the most predictive angiographic value of unsuccessful PCI, however emerging new techniques have allowed for acceptable rates of success and lower rates of complications [20, 29]. The three most common wiring techniques are antegrade wire escalation, antegrade dissection and re-entry, and the retrograde approach (Fig. 2, Ref. [30]). The most well-recognized formula for incorporating these techniques is the Hybrid approach developed as a consensus from several high-volume CTO operators [31]. The Hybrid approach determines which technique to employ based on four characteristics of dual catheter angiography: the morphology of proximal cap, the occlusion length, the distal vessel size and presence of bifurcations beyond the distal cap, and the location and suitability of retrograde conduits. This algorithm favors the antegrade approach for shorter lesions, antegrade dissection and re-entry for longer lesions, and the retrograde approach in lesions with ambiguous proximal caps, poor distal targets and strong collaterals. A reverse dissection and re-entry can be used if the retrograde approach is unsuccessful [31]. This approach has been validated by several observational studies with a high success rate and low complications [32]. Detailed description of highly specialized techniques is beyond the scope of this review.

## 2. Percutaneous Treatment Strategies to Saphenous Vein Graft

Saphenous vein grafts (SVGs) are often times used as a conduit during CABGs (coronary artery bypass graft) and are associated with poor patency rates [33, 34]. Generally, PCI is preferred to repeat CABG because of the high rate of perioperative complications [35]. PCI intervention of SVG has a lower success rate, worse short term and long term outcomes, and higher rates of slow and no-reflow phenomena compared to native coronary artery revascularization [36, 37, 38, 39]. In many ways SVG intervention is dissimilar to native coronary artery intervention. Arterialization of vein grafts can lead to poor outcomes due to intimal hyperplasia, which consists of thin-capped atherosclerotic plaques that are diffuse and prone to embolization [40, 41, 42]. For these reasons ESC guidelines recommend consideration of a PCI to native coronary arteries before considering SVG PCI [13].

### 2.1 Stent Selection 

It was first shown that PCI to SVG had favorable procedural success rates and lower rates of MACE compared to balloon angioplasty in the bare metal stent (BMS) era [43]. More recently, however, there has been debate over the use of drug eluting stents (DES) compared to BMS for SVG intervention. BMS have a hypothetical advantage of larger diameters. Early trials showed a higher all-cause mortality at 3 years with the currently obsolete first generation sirolimus-eluting stent compared to BMS [44]. Subsequent trials, however, illustrated an improvement in MACE at 1 year with first generation DES compared to BMS driven by a reduction in target lesion revascularization. This may be negated by late catch up events and subsequent revascularizations ultimately leading to similar outcomes between the two stent types [45, 46].

In the modern era, trials have shown second generation DES to have no differences compared to BMS [47]. It is speculated that second generation DES do not display the same benefit, as compared to BMS, in SVG lesions as they do in native coronary lesions due to the difference in pathophysiology of the underlying disease. Venous grafts are more susceptible to rapidly progressing atherosclerotic disease and once a graft starts degenerating there is a high rate of failure regardless of the intervention [48]. Additionally, while the antiproliferative effect of DES may be effective within the segment, they are unable to treat segments outside of the stented area that are often the cause of long term graft failure [48].

Multiple meta-analyses have investigated the differences between DES and BMS in this setting. Despite somewhat mixed results, most do find DES to be superior to BMS [48, 49, 50, 51, 52]. The 2011 ACC/AHA/SCAI guidelines have listed stent selection in PCI to an SVG as a clinical determination with a slight preference for DES [53]. While the 2021 ACC/AHA/SCAI guidelines do not specifically reference stent selection in PCI to SVG, it does recommend DES over BMS in all PCI [12]. Nevertheless, BMS are an acceptable alternative in specific situations such as low income settings.

### 2.2 Embolic Protection Devices

Embolic protection devices (EPD) catch atheroembolic plaques that may dislodge during SVG intervention. Despite ACC/AHA/SCAI recommendations supporting their use, these devices remain underutilized [12, 53]. Their limited use has been supported by some retrospective data that suggest their limited efficacy [54, 55]. Other barriers include their high costs, necessary training and lack of uniform practices [54, 55].

These devices are broken into three categories: distal occlusion aspiration devices, distal embolic filters and proximal occlusion aspiration devices. Distal embolic devices were both the first to be developed and still account for a significant majority of use in the contemporary era [54]. The SAFER (Saphenous Vein Graft Angioplasty Free of Emboli Randomized) trial was the first to evaluate EDP efficacy and showed a reduction in no-reflow events, MI (myocardial infarction) and 30-day MACE with a distal balloon occlusion and aspiration system compared to conventional guidewires [56]. Distal filters have the potential benefit of maintaining perfusion during procedures and many trials have shown them to be non-inferior compared to the distal balloon occlusion device [57, 58, 59, 60]. Proximal occlusion devices are beneficial if there is no distal landing zone. Although they have been shown to be non-inferior compared to distal occlusion devices, they are no longer manufactured [61].

Given the present controversy surrounding EPDs, further research is needed to identify in which situations EPDs are warranted as well as re-evaluation of these devices in the RCT setting in the contemporary era. Cardiology societies can help to establish universal guidelines to improve skill, expertise and outcomes.

## 3. In Stent Restenosis

Intracoronary stent restenosis (ISR) is the progressive luminal re-narrowing of stented segments, or immediately adjacent area, by >50% of stent diameter [62]. The pathophysiology of these lesions is related to both (1) biologic processes, which include intimal injury and neointimal formation in BMS and chronic inflammation and neoatherosclerosis in DES, as well as (2) mechanical processes, including stent fracture, stent under-expansion, stent malappostion, suboptimal stent size and uncovered stent struts [63, 64]. This phenomenon poses a unique challenge for interventionalists as there are multiple causes and heterogenous patterns of disease that require a specialized and unique approach to each individual lesion. Fortunately, these types of lesions have dramatically decreased since the development of DES and currently only occur in roughly 5–10% of PCI [64, 65]. ISR typically presents within the first year of stent implementation and can present as angina or new ACS [64].

Systematic classification of ISR patterns in BMS includes (a) focal, (b) diffuse, (c) diffuse with extension outside of the stent margin, and (d) occlusive [66]. This simple system has proven helpful in predicting revascularization success, with improved outcomes in focal compared to non-focal lesions, and remains effective in the DES era [67]. An alternative system that categorizes lesions based on mechanism and characteristics of failure may also be helpful for determining intervention: mechanical (Type I), biologic (Type II), mixed (Type III), CTO (Type IV) or previously treated ISR with >2 stents (Type V) [63].

The treatment of ISR is dependent on the underlying cause, the characteristics of the stenosis and the type of stent initially placed. The use of intracoronary imaging such as IVUS (intravascular ultrasound) and OCT (optical coherence tomography) is recommended as it can help identify the underlying cause and pattern of stenosis as well as improve outcomes [63, 64]. IVUS has been shown to be able to detect neo-intimal hyperplasia obstructing the stent, stent under-expansion and edge problems. Due to its superior axial resolution, OCT is able to provide greater detailed images of the vessel-lumen interface and neointimal tissue [65]. Specific findings may determine which tools are needed for adjunctive therapy. For instance calcifications may require atherectomy, stent under-expansion may require high pressure balloons, and neointimal hyperplasia may require cutting or scoring or drug coated balloons (DCB) [63]. Additionally, intravascular imaging provides the operator greater detail of the number of stents in place in the scenario of repeated in stent restenosis [63].

In cases of ISR due to stent under-expansion, high pressure balloons are the standard of care and atherectomy or lithotripsy adjuncts can be considered [68]. Cutting or scoring balloons are additional tools that may be used for ISR caused by neointimal hyperplasia and have been found to outperform standard balloons [69]. More recent RCTs, however, have shown them to be inferior when compared to repeat DES for focal restenosis due to neointimal hyperplasia [70].

Vascular brachytherapy may be offered as a last resort therapy because it can inhibit neointimal formation within the stent by delivering intracoronary radiation [71, 72, 73]. High rates of late restenosis have led to the decline of this modality [64]. Its use is limited to patients who have recurrent ISR at sites with >2 stents and are not amenable to repeat PCI, are not candidates to undergo CABG, and are not in areas where DCB are available [12, 68].

The most popular option for ISR management remains repeat DES intervention, particularly in focal ISR without stent under-expansion. First generation DES were effective in treatment of BMS ISR in multiple trials [74, 75]. A switch strategy using a different first generation DES to treat DES ISR has also been considered given that the etiology for DES restenosis may be due to drug resistance; however, results were rather mixed and inconclusive [76, 77].

This debate has largely fallen to the wayside since the development of second generation DES. Trials proved that Everolimus-eluting stents are efficacious and superior to DCB for the treatment of BMS ISR and DES ISR [78, 79, 80, 81]. Additional analyses have shown that second generation stents are superior to first generation stents for both BMS and DES ISR [82].

Finally, another option is DCB, which use lipophilic drugs, such as Paclitaxel, to inhibit neointimal formation. Presently they only have limited availability in the United States, however they can be found commonly worldwide and have been shown to be superior to standard balloons [83].

A 2015 and 2016 meta-analyses with 5923 and 7474 subjects, respectively, have demonstrated second generation DES, followed by DCB, to be the most effective treatment modality for ISR compared to balloon angioplasty, brachytherapy, BMS and rotational atherectomy with fewer target lesion revascularization and improved diameter stenosis at angiographic follow up [84, 85]. With this evidence, the 2018 ESC/EACTS guidelines support the use of both second generation DES and DCB for ISR [13].

## 4. Antiplatelet Strategies in Complex Lesions

One unifying component of this heterogenous group of complex lesions is the recognition that they carry a higher risk of ischemic events. While dual antiplatelet therapy (DAPT) with aspirin and P2Y12 inhibitors remains the backbone for medical therapy following deployment of DES, the optimal duration and combination of medications is still evolving. A meta-analysis of 6 RCTs comparing shorter or longer DAPT found that complex PCI was associated with higher rates of MACE and coronary thrombotic events and that longer duration of DAPT in complex PCI was associated with lower rates of MACE. It also found that complex PCI was not associated with increased bleeding compared to non-complex PCI [86].

While this analysis identified complex lesions as an important driver of future ischemic events attenuated by prolonged DAPT, it also illustrated an increased risk of bleeding with prolonged DAPT in all-comers. Further analyses may clarify which patients benefit from extended DAPT (and for how long a period), and for which the bleeding risk outweighs this benefit.

One option includes detailed risk stratification with scoring systems. The PRECISE-DAPT (Predicting Bleeding Complications in Patients Undergoing Stent Implantation and Subsequent Dual Antiplatelet Therapy) score is a validated system intended to predict bleeding outcomes to help determine DAPT duration at the time of intervention [87]. Subgroup analysis of complex PCI from PRECISE-DAPT showed that extended DAPT (12–24 months) was superior to short DAPT only in those with low bleeding risk (PRECISE-DAPT <25) [88]. Without any benefit in ischemic outcomes in those with high bleeding risk and complex lesions, bleeding risk- not lesion complexity- should be the driver in decision making for prolonging DAPT.

The PARIS (Patterns of Non-Adherence to Anti-Platelet Regimen in Stented Patients) scores are alternatives to the PRECISE-DAPT that uses patients clinical characteristics, and not procedural characteristics, to predict both bleeding and thrombotic risks. This study did not find complex procedures to be predictive of either thrombotic events or major bleeding [89].

The DAPT (Dual Antiplatelet Therapy) score was developed with the intention to guide clinicians on whether to prolong DAPT after completion of 12 months of DAPT without a sentinel event [90]. Subgroup analysis from the original study showed that within the first year there is a relative increase in MI or stent thromboses in those with complex intervention compared to non-complex intervention but thereafter the risk is similar. This highlights that risks of ischemic events are not static over time but rather dynamic. Complex PCI is a risk factor for earlier ischemic events; within the first year in particular [91].

Novel approaches have been developed to limit bleeding risk while protecting against ischemic events by de-escalating from DAPT to P2Y12 monotherapy. This approach has been shown to be efficacious for those presenting with both stable CAD as well as ACS [92, 93, 94, 95, 96].

Post-hoc analyses of patients with complex angiographic features from the major trials investigating this approach showed similar results (Table 1). In TWILIGHT-Complex (Ticagrelor with Aspirin or Alone in High-Risk Patients after Coronary Intervention) there was no significant difference in ischemic outcomes however there was less major and minor bleeding in those with complex angiographic features who were treated with abbreviated DAPT followed by P2Y12 monotherapy as compared to prolonged DAPT [97]. In the GLOBAL LEADERS subgroup analysis of patients with complex angiographic features there was a significant reduction in the primary endpoint of all cause death or MI with no change in bleeding risk [98]. The STOPDAPT-2 (Short and Optimal Duration of Dual Antiplatelet Therapy After Everolimus-Eluting Cobalt-Chromium Stent) subanalysis showed benefit in the primary outcome of combined ischemic and bleeding events in the cohort of patients with complex angiographic features who received shortened DAPT followed by P2Y12 monotherapy as compared to extended DAPT [99]. The TICO (Ticagrelor Monotherapy After 3 Months in the Patients Treated With New Generation Sirolimus-eluting Stent for Acute Coronary Syndrome) subgroup analysis that only included those with complex angiographic features found no difference in both ischemic and bleeding outcomes [100]. SMART-CHOICE (Smart Angioplasty Research Team: Comparison Between P2Y12 Antagonist Monotherapy vs Dual Antiplatelet Therapy in Patients Undergoing Implantation of Coronary Drug-Eluting Stents) was yet another trial whose subgroup analysis for complex angiographic anatomy showed no difference in both ischemic and bleeding outcomes [101].

**Table 1.** **Summary of complex PCI substudies from randomized controlled trials investigating abbreviated DAPT followed by P2Y12 monotherapy**.

Complex PCI substudy	Antiplatelet strategy		Ischemic Primary Outcomes		Bleeding Primary Outcomes	
	Abbreviated	Prolonged	Abbreviated	Prolonged	Abbreviated	Prolonged
Twilight *	3 months DAPT → 12 months Ticagrelor	12 months DAPT	43 (3.8%)	56 (4.9%)	48 (4.2%)	90 (7.7%)
n = 2342	(n = 1158)	(n = 1184)	HR = 0.77		HR = 0.54	
			(95% CI 0.52–1.15)		(95% CI 0.38–0.76)	
Global Leaders †	1 month DAPT → 23 months Ticagrelor	12 months DAPT → 12 months Aspirin	80 (3.5%)	124 (5.4%)	55 (2.4%)	57 (2.5%)
n = 4570	(n = 2283)	(n = 2287)	HR = 0.64		HR = 0.97	
			(95% CI 0.48–0.85)		(95% CI 0.67–1.40)	
STOP-DAPT 2 ‡	1 month DAPT → Clopidogrel	12 months DAPT	4 (1.7%)	8 (3.0%)	0 (0.0%)	6 (2.3%)
n = 509	(n = 245)	(n = 264)	HR = 0.54		—	
			(95% CI 0.16–1.79)			
TICO §	3 months DAPT → 9 months Ticagrelor	12 months DAPT	7 (2.6%)	12 (4.9%)	5 (1.9%)	9 (3.6%)
n = 517	(n = 270)	(n = 247)	HR = 0.52		HR = 0.50	
			(95% CI 0.21–1.33)		(95% CI 0.17–1.49)	
SMART-CHOICE ¶	3 months DAPT → 9 months P2Y12	12 months DAPT	10 (3.8%)	10 (4.2%)	5 (1.9%)	8 (3.4%)
n = 498	(n = 260)	(n = 238)	HR = 0.92		HR = 0.58	
			(95% CI 0.38–2.21)		(95% CI 0.19–1.77)	

(*) Primary ischemic outcome was death, MI or stroke. Primary bleeding outcome was BARC type 2, 3 or 5 bleeding. (†) Primary ischemic outcome was death or MI. Primary bleeding outcome was BARC type 3 or 5 bleeding. (‡) Primary ischemic outcome was cardiovascular death, MI, stent thrombosis or stroke Primary bleeding outcome was TIMI major or minor bleeding. § Primary ischemic outcome was all-cause death, MI, ST, stroke, or TVR. Primary bleeding outcome was TIMI major bleeding. ¶ Primary ischemic outcome was all-cause death, MI or stroke. Primary bleeding outcome was BARC 2–5.

Two recent trials, MASTER-DAPT (The Management of High Bleeding Risk Patients Post Bioresorbable Polymer Coated Stent Implantation with an Abbreviated versus Standard DAPT Regimen) and STOPDAPT-2 ACS have been published on this topic [102, 103]. MASTER-DAPT again showed that abbreviated DAPT had non-inferior thrombotic outcomes and reduced bleeding events in those with high bleeding risk [102]. STOPDAPT-2 ACS failed to reach non-inferiority for the abbreviated DAPT strategy in those who presented with ACS [103]. Neither study has yet to publish complete data on outcomes specifically within the study population who had complex angiographic features. The STOPDAPT-2 ACS trial included subgroup analysis of those who had certain complex angiographic features, such as patients who were treated with total stent length ≥28 mm and with 2+ target vessels. For these two subgroups there were no differences between abbreviated DAPT and traditional DAPT in both thrombotic and bleeding outcomes [103].

It is worth noting that these sub-studies were not powered to answer the question of the optimal antiplatelet treatment in those with complex PCI. Furthermore, while these sub-studies included patients exclusively with complex angiographic features, each study used slightly different definitions for angiographic complexity. Amongst these subgroup analyses, the number of patients included with complex procedural characteristics ranged between 14.9–32.9% of the original studies [97, 98, 99, 100, 101]. Additionally the study populations are not completely comparable. The TWILIGHT trial exclusively used patients with high risk of bleeding, the TICO trial evaluated patients exclusively with ACS and the STOPDAPT-2 trial included a large majority of patients with low to intermediate bleeding risk [92, 94, 96]. Nevertheless, these antiplatelet strategies appear to be safer, equally efficacious and simpler compared to the traditional DAPT approach.

## 5. Future Directions and Conclusion

Complex PCI has evolved since its initial description in 1985. What started as a representation of rudimentary morphologic features now includes complex angiographic characteristics including calcifications, in stent restenosis, SVG intervention, chronic total occlusions, bifurcation lesions as well as left main disease. Many strides have been made in the field that have allowed interventionalists to perform intricate procedures on bifurcation lesions and new technologies have helped intervene on calcified, stenotic and occluded lesions. Future trials should better characterize which approach to take for bifurcation lesions, which CTOs require intervention, what tools are best in ISR and the utility of embolic devices in SVG intervention. To further our knowledge on this broad topic it is time we rally around the definition put forth by SCAI to allow us to study these lesions in a more consistent manner and rationalize results in a more clinically meaningful way.

## Abbreviations

ACC, American College of Cardiology; ACS, Acute Coronary Syndrome; AHA, American Heart Association; BMS, Bare Metal Stent; CABG, Coronary Artery Bypass Graft; CAD, Coronary Artery Disease; CMR, Cardiac Magnetic Resonance Imaging; PCI, Percutaneous Intervention; CTO, Chronic Total Occlusion; DCB, Drug Coated Balloons; DECISION-CTO, Drug-Eluting Stent Implantation Versus Optimal Medical Treatment in Patients With Chronic Total Occlusion; DES, Drug Eluting Stent; EACTS, European Association for Cardiothoracic Surgery; EPD, Embolic Protection Device; ESC, European Society of Cardiology; EURO-CTO, Evaluate the Utilization of Revascularization or Optimal Medical Therapy for the Treatment of Chronic Total Coronary Occlusions; EXPLORE, Evaluating Xience and Left Ventricular Function in Percutaneous Coronary Intervention on Occlusions After ST-Elevation Myocardial Infarction; ISCHEMIA, International Study of Comparative Health Effectiveness with Medical and Invasive Approaches; ISR, Intracoronary Stent Restenosis; IVUS, Intravascular Ultrasound; LAD, Left Anterior Descending artery; LGE, Late Gadolinium Enhancement; LMCAD, Left Main Coronary Artery Disease; MACE, Major Adverse Cardiovascular Events; MASTER-DAPT, The Management of High Bleeding Risk Patients Post Bioresorbable Polymer Coated Stent Implantation with an Abbreviated versus Standard DAPT Regimen; MI, Myocardial Infarction; MPI, Myocardial Perfusion Imaging; OCT, Optical Coherence Tomography; PARIS, Patterns of Non-Adherence to Anti-Platelet Regimen in Stented Patients; PCI, Percutaneous Intervention; PET, Positron Emission Tomography; PRECISE-DAPT, Predicting Bleeding Complications in Patients Undergoing Stent Implantation and Subsequent Dual Antiplatelet Therapy; RCT, Randomized Controlled Trial; REVASC, Recovery of Left Ventricular Function After Stent Implantation in Chronic Total Occlusion of Coronary Arteries; SAFER, Saphenous Vein Graft Angioplasty Free of Emboli Randomized; SCAI, Society for Cardiovascular Angiography and Intervention; SPECT, Single Photon Emission Computed Tomography; SMART-CHOICE, Smart Angioplasty Research Team, Comparison Between P2Y12 Antagonist Monotherapy vs Dual Antiplatelet Therapy in Patients Undergoing Implantation of Coronary Drug-Eluting Stents; STEMI, ST segment elevation myocardial infarction; STOPDAPT-2, Short and Optimal Duration of Dual Antiplatelet Therapy After Everolimus-Eluting Cobalt-Chromium Stent; SVG, Saphenous Vein Grafts; SYNTAX, Synergy between PCI with Taxus and Cardiac Surgery; TICO, Ticagrelor Monotherapy After 3 Months in the Patients Treated With New Generation Sirolimus-eluting Stent for Acute Coronary Syndrome; TIMI, Thrombolysis in Myocardial Infarction; TWILIGHT, Ticagrelor with Aspirin or Alone in High-Risk Patients after Coronary Intervention.
